# Inferring the Minimal Genome of *Mesoplasma florum* by Comparative Genomics and Transposon Mutagenesis

**DOI:** 10.1128/mSystems.00198-17

**Published:** 2018-04-10

**Authors:** Vincent Baby, Jean-Christophe Lachance, Jules Gagnon, Jean-François Lucier, Dominick Matteau, Tom Knight, Sébastien Rodrigue

**Affiliations:** a Département de Biologie, Université de Sherbrooke, Sherbrooke, Québec, Canada; b Ginkgo Bioworks, Boston, Massachusetts, USA; University of California, Berkeley

**Keywords:** *Mesoplasma florum*, comparative genomics, minimal genome, transposon mutagenesis

## Abstract

The creation and comparison of minimal genomes will help better define the most fundamental mechanisms supporting life. Mesoplasma florum is a near-minimal, fast-growing, nonpathogenic bacterium potentially amenable to genome reduction efforts. In a comparative genomic study of 13 M. florum strains, including 11 newly sequenced genomes, we have identified the core genome and open pangenome of this species. Our results show that all of the strains have approximately 80% of their gene content in common. Of the remaining 20%, 17% of the genes were found in multiple strains and 3% were unique to any given strain. On the basis of random transposon mutagenesis, we also estimated that ~290 out of 720 genes are essential for M. florum L1 in rich medium. We next evaluated different genome reduction scenarios for M. florum L1 by using gene conservation and essentiality data, as well as comparisons with the first working approximation of a minimal organism, Mycoplasma mycoides JCVI-syn3.0. Our results suggest that 409 of the 473 M. mycoides JCVI-syn3.0 genes have orthologs in M. florum L1. Conversely, 57 putatively essential M. florum L1 genes have no homolog in M. mycoides JCVI-syn3.0. This suggests differences in minimal genome compositions, even for these evolutionarily closely related bacteria.

**IMPORTANCE** The last years have witnessed the development of whole-genome cloning and transplantation methods and the complete synthesis of entire chromosomes. Recently, the first minimal cell, Mycoplasma mycoides JCVI-syn3.0, was created. Despite these milestone achievements, several questions remain to be answered. For example, is the composition of minimal genomes virtually identical in phylogenetically related species? On the basis of comparative genomics and transposon mutagenesis, we investigated this question by using an alternative model, Mesoplasma florum, that is also amenable to genome reduction efforts. Our results suggest that the creation of additional minimal genomes could help reveal different gene compositions and strategies that can support life, even within closely related species.

## Abstract (precis)

The last years have witnessed the development of whole-genome cloning and transplantation methods and the complete synthesis of entire chromosomes. Recently, the first minimal cell, Mycoplasma mycoides JCVI-syn3.0, was created. Despite these milestone achievements, several questions remain to be answered. For example, is the composition of minimal genomes virtually identical in phylogenetically related species? On the basis of comparative genomics and transposon mutagenesis, we investigated this question by using an alternative model, Mesoplasma florum, that is also amenable to genome reduction efforts. Our results suggest that the creation of additional minimal genomes could help reveal different gene compositions and strategies that can support life, even within closely related species.

## INTRODUCTION

Synthetic genomics is an emerging field of synthetic biology combining different approaches and technologies to chemically synthesize sections of chromosomes or even entire genomes (1, 2), thus enabling the generation of engineered organisms that significantly differ from those found in nature. Given sufficient knowledge and proper execution, this could lead to the rational design of organisms built to accomplish specific tasks (3). However, the complexity of current model organisms is overwhelming and outstrips our ability to understand how cells operate on a global scale (4). Minimal genomes, in addition to providing invaluable information about the essential genes and fundamental principles required to sustain life, would therefore facilitate systematic investigations toward a global understanding of cell functioning. Minimal cells could also become interesting platforms for rapid and affordable prototyping of engineered cells, further helping to uncover underlying genome design rules. So far, three main approaches have been used to determine the minimal gene set in various organisms: comparative genomic analyses, gene inactivation studies, and progressive genome reduction.

Comparative genomics uses sequence-based strategies to identify conserved genes, which are hypothesized to be maintained throughout evolution and shared across different organisms because of their contribution to cell fitness (5). The exact number and nature of conserved genes have been found to vary considerably between studies, depending on the phylogenetic distribution (6) and number of genomes analyzed (5, 7). For example, Land and colleagues reported that 3,188 genes were always detected in Escherichia coli (8) while only 38 genes were found to be shared by 147 different species of bacteria and archaea (6). Some conserved gene sets are thus certainly too large to reveal the minimal genome and rather correspond to important functions that are not necessarily essential but likely contribute to the fitness of an organism in its natural habitat (9). Other gene sets are simply too small to support basic functions like replication, transcription, and translation. The results obtained through comparative genomics approaches are thus highly dependent on the set of organisms analyzed.

Genes that are conserved within a species are thought to be important or essential in their natural environment. However, laboratory and environmental conditions can greatly differ, resulting in different genetic requirements. Experimental assessment of essential genes can be achieved through individual gene inactivation studies. In this regard, gene deletion (9, 10), transposon mutagenesis (11, 12), and transcriptional interference (13, 14) were used to identify dispensable genes. The results obtained from such experiments depend largely on growth conditions since, for example, cells may need certain metabolic pathways unless their products are already available in the medium (15). Certain genes are essential only in the presence of another gene, for example to balance or counteract the activity of another gene product. For example, the antitoxin of a toxin/antitoxin system is only essential while the toxin is also present (9). At lower insertion densities, transposon mutagenesis is likely to overestimate the number of essential genes because of the higher probability of missing genes simply by chance (16). On the other hand, gene inactivation strategies can overestimate the number of dispensable genes since duplicated sequences or alternate metabolic pathways may be interrupted individually but not simultaneously, a phenomenon called synthetic lethality (17). Overall, these phenomena can lead to biases and uncertainties in the estimation of the number of essential genes.

Cumulative gene deletions that result in genome reduction provide a more accurate picture of possible minimal genome compositions of a given organism. However, this approach involves considerable effort along with well-developed genetic tools. So far, this strategy has only been applied to a few organisms, including E. coli (18–21), Bacillus subtilis (22), Streptomyces avermitilis (23), Pseudomonas putida (24), and Mycoplasma mycoides subsp. capri (2). The latter organism has undergone the most drastic streamlining, with the removal of ~50% of its original genome, resulting in the creation of M. mycoides JCVI-syn3.0 containing a single chromosome of 531 kbp. M. mycoides JCVI-syn3.0 was described as the first “working approximation of a minimal cell” (2) and is currently the simplest organism capable of autonomous growth in axenic culture. Interestingly, minimal genome designs initially proposed for M. mycoides based on single gene inactivation by transposon mutagenesis and other literature-based knowledge were not viable (2). Many optimization and debugging steps were required to obtain M. mycoides JCVI-syn3.0, highlighting the difficulty of identifying and understanding the roles of essential genes, even in the simplest cells.

Mesoplasma florum is a bacterium first isolated from a lemon tree flower in 1984 (25). Unlike many other members of the class Mollicutes, M. florum shows a short doubling time of <40 min, requires no sterol for growth, and has no known pathogenic potential. The genomes of two M. florum strains, L1 and W37 (26), have been completely sequenced, revealing a single circular chromosome of ~800 kbp and positioning this species among the simplest free-living organisms. Basic genetic manipulation tools comprising antibiotic resistance genes, plasmids, and transformation methods have recently been developed for M. florum (27). Furthermore, the complete genome of M. florum L1 has also been cloned in yeast and transplanted into a recipient Mycoplasma capricolum subsp. capricolum strain (28, 29), which will enable sophisticated modifications and reengineering of the M. florum chromosome. This combination of low cell complexity, ease of manipulation, and the availability of genome engineering methods makes M. florum an interesting model for systems biology and synthetic genomics.

Here, we report a comparative genomic analysis of 13 M. florum strains. These data were investigated in conjunction with transposon mutagenesis to identify conserved, accessory, and essential genes in this species. We also discuss different scenarios for eventual M. florum genome reduction efforts according to results presented here and using comparisons with the phylogenetically related strain M. mycoides JCVI-syn3.0.

## RESULTS

### Genome sequencing of 11 M. florum strains.

**FIG 1:**
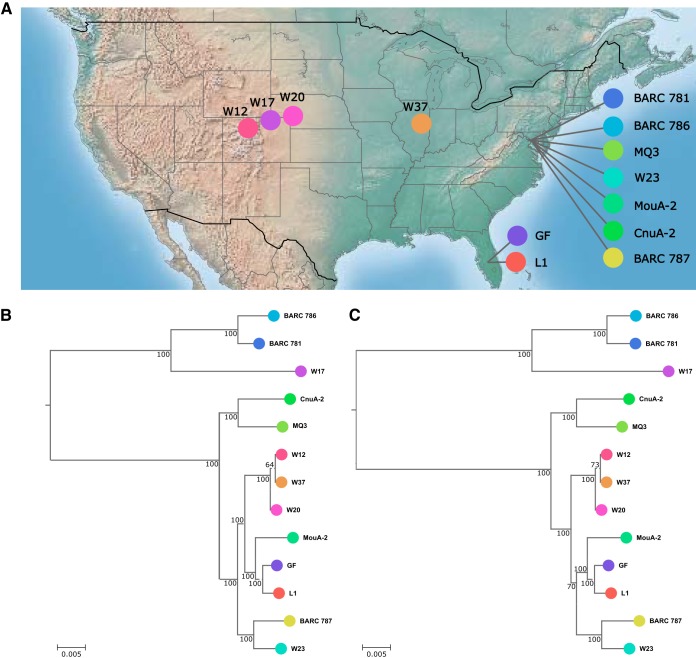
M. florum strain sampling and phylogeny. (A) Isolation sites of the 13 M. florum strains analyzed in this study. (B, C) M. florum phylogenetic trees constructed by using concatenated alignments of 412 conserved proteins and the Kimura distance model (B) or maximum likelihood (C). M. capricolum was used as the outgroup for both trees and is not shown because of the long branch length. Bootstrap values correspond to 100 repetitions. In both trees, branch length represents the substitution rate per site per unit of alignment length.

Several *Mesoplasma* species have been isolated from plants or insects since the 1980s (25, 30–34). Of these, we have obtained 13 M. florum strains available from culture collections (Table 1). Two strains originated in Florida, and 11 were collected on a longitudinal transect from Maryland to Colorado (Fig. 1A). This variety of environments, host organisms, and climates was expected to result in diversity of gene content in the genomes analyzed. The complete genome sequences of M. florum strains L1 and W37 have previously been reported (26). The genomes of the remaining 11 M. florum strains were sequenced by using a combination of the Illumina, Pacific Biosciences, and Sanger technologies. In total, seven genomes (L1, W37, BARC 787, MQ3, CnuA-2, MouA-2, and W23) were fully assembled, resulting in circular chromosomes without any ambiguous positions. The genomes of BARC 786, BARC 781, GF, W17, W20, and W12 were not unequivocally resolved during their assembly (containing one to four gaps) because of a total of five distinct repeated elements, either duplications or tandem repeats (Table 1). By keeping at least one copy of each problematic region in each final assembly, we estimate that virtually all genes are represented in these genomes and that no more than a few kilobase pairs are missing from the chromosomes of these strains. The genome size of the 13 sequenced strains was found to vary between 738,512 bp (BARC 787) and 830,640 bp (20), with a mean of 794.5 ± 25.6 kbp. As expected from the genome sequences of other members of the class Mollicutes (35), the GC content of every strain analyzed was found to be relatively low, with an average of 27.1% ± 0.2% (Table 1).

**TABLE 1:** M. florum strains and genome sequencing overview

Strain	GenBank accession no.	Genome size (bp)	% GC	No. of gaps	No. of protein- coding genes	No. of functional RNAs	No. of accessory genes	Genome coding percentage	Collection	Source	Reference or source
L1	AE017263.1	793,224	27.0	0	685	35	136	93.9	Florida	*Citrus limon*	25
W37	CP006778.1	825,824	27.0	0	731	35	179	93.3	Gibson City, IL	*Solidago* sp.	32
BARC 787	CP022514	738,512	27.1	0	651	35	102	94.0	Beltsville, MD	Unspecified insect	Unpublished^*a,d*^
MQ3	CP022512	793,277	27.0	0	698	35	146	94.2	Maryland	*Monobia quadriens*	34
CnuA-2	CP022513	813,801	27.0	0	710	35	158	93.9	Maryland	*Coleoptera*: *Cantharidae*	33
MouA-2	CP022508	781,099	27.0	0	685	35	134	93.5	Beltsville, MD	Vespid wasp	Unpublished^*a,e*^
W23	CP022505	773,885	27.1	0	688	35	137	94.1	North Platte, NE	Helianthus annuus	32
BARC 786	CP022510	765,660	27.4	1^b^	669	35	119	93.5	Beltsville, MD	Beetle	Unpublished^*a,d*^
BARC 781	CP022511	803,948	27.1	1^b^	691	35	139	92.9	Beltsville, MD	Beetle	Unpublished^*a,d*^
GF	CP022509	792,347	27.0	2^b^,^c^	699	35	147	94.0	Florida	Citrus paradisi	25
W17	CP022507	787,107	27.4	3^c^	693	35	140	92.6	Pawnee National Grassland, CO	*Aster* sp.	32
W20	CP022506	830,640	27.0	4^b^,^c^	740	35	187	92.5	Ogallala, NE	*Aster simplex*	32
W12	CP022432	829,202	27.0	4^b^,^c^	734	35	181	93.3	Kremmling, CO	Chrysothamnus sp.	32

a Gail E. Gasparich and Robert E. Davis, personal communication.

b Gaps containing tandem repeats.

c Gaps linked to gene duplications.

d Isolated by R. Whitcomb in 1986.

e Isolated by T. Clark and K. Hackett in 1986.

### Genome annotation and phylogenetic analysis.

To apply a uniform procedure and minimize potential biases between annotations, gene prediction was performed for all of the M. florum genomes studied, including those of the previously sequenced L1 and W37 strains (see Data Sets S1 and S2 in the supplemental material, respectively). Every genome was predicted to contain 29 tRNA genes, as well as two rRNA gene loci, each encoding 5S, 16S, and 23S rRNAs. Between 651 and 740 protein-coding genes were predicted, depending on the M. florum strain analyzed, for a total of 9,074 putative proteins in the combined 13 genomes. The genomes showed a protein-coding gene to kilobase pair ratio of ~0.88, which is typical of bacterial genomes (36). All proteins were clustered on the basis of sequence homology (37). Genes conserved among all M. florum strains and M. capricolum as the outgroup were used to construct phylogenetic trees based on the Kimura distance model (38) (Fig. 1B) and maximum likelihood (39) (Fig. 1C). The two trees showed practically identical branch lengths and very similar topologies, diverging only slightly around the L1/GF/MouA-2 subgroup. Overall, the trees revealed two main groups that are, in turn, subdivided into smaller phylogenetic clusters. Although some closely related strains were isolated from nearby locations or similar organisms, the geographic origin and potential host were not sufficient to explain the phylogeny observed.

10.1128/mSystems.00198-17.1 DATA SET S1  Summary of *M. florum* L1 gene information. Download DATA SET S1, XLSX file, 0.1 MB. Copyright © 2018 Baby et al. 2018 Baby et al. This content is distributed under the terms of the Creative Commons Attribution 4.0 International license.

10.1128/mSystems.00198-17.2 DATA SET S2  *M. florum* genome annotations. Download DATA SET S2, XLSX file, 4.2 MB. Copyright © 2018 Baby et al. 2018 Baby et al. This content is distributed under the terms of the Creative Commons Attribution 4.0 International license.

**FIG 2:**
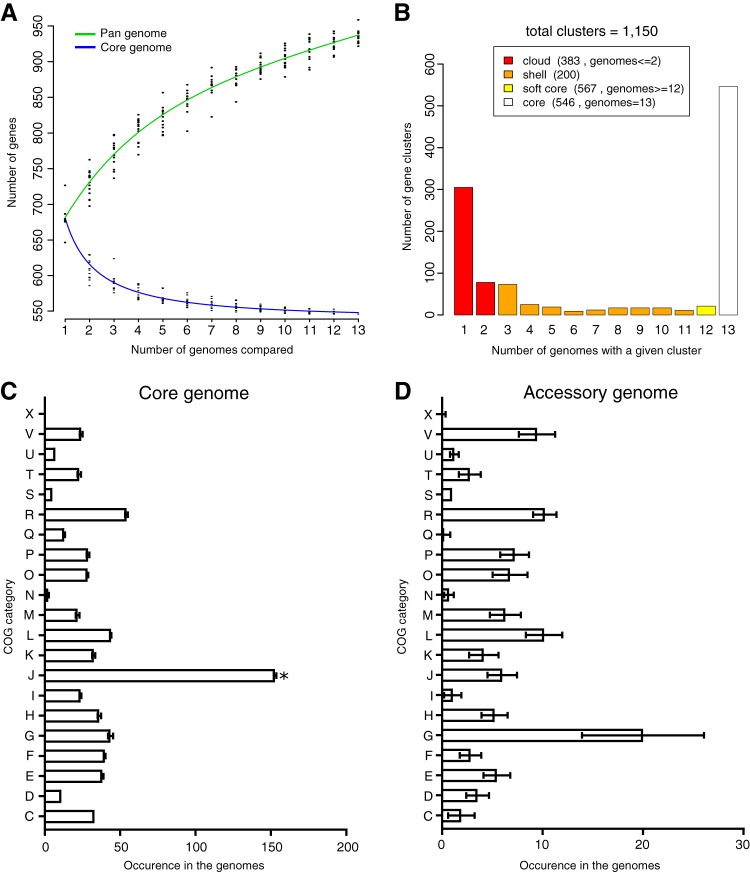
Pangenomes and core genomes of 13 M. florum strains. (A) Gene number estimation curves for the core genomes (blue, bottom curve) and pangenomes (green, top curve) were generated by the methods of Willenbrock et al. (60) and Tettelin et al. (61), respectively. (B) Prevalence of the different protein clusters across 13 strains. (C, D) Average number of protein groups in COG categories found in the core (C) and accessory (D) genomes of each strain. The COG categories are as follows: C, energy production and conversion; D, cell cycle control, cell division, and chromosome partitioning; E, amino acid transport and metabolism; F, nucleotide transport and metabolism; G, carbohydrate transport and metabolism; H, coenzyme transport and metabolism; I, lipid transport and metabolism; J, translation, ribosomal structure, and biogenesis; K, transcription; L, replication, recombination, and repair; M, cell wall/membrane/envelope biogenesis; N, cell motility; O, posttranslational modification, protein turnover, chaperones; P, inorganic ion transport and metabolism; Q, secondary metabolite biosynthesis, transport, and catabolism; R, general function prediction only; S, function unknown; T, signal transduction mechanisms; U, intracellular trafficking, secretion, and vesicular transport; V, defense mechanisms; X, mobilome (prophages, transposons).

The predicted proteins in the M. florum strains were grouped into a total of 1,150 homologous gene clusters (Data Set S3). A core set of 546 clusters was observed in all representatives, resulting in the conservation of approximately 80% of the protein-coding genes in every strain (Fig. 2A and B). A majority (75.5%) of the 604 remaining gene clusters, also called the accessory genome or pangenome, was found in no more than three strains. Additionally, the number of gene cluster families discovered kept increasing as a function of the number of strains analyzed (Fig. 2A), suggesting that M. florum has an open pangenome and that gene diversity in this species was not fully explored by investigating these 13 genomes. Considering that an average of 23.5 gene families were found to be unique in each M. florum strain, several additional gene cluster families should be found simply by incorporating more genomes into the analysis.

10.1128/mSystems.00198-17.3 DATA SET S3  *M. florum* gene cluster families used in this study. Download DATA SET S3, XLSX file, 0.1 MB. Copyright © 2018 Baby et al. 2018 Baby et al. This content is distributed under the terms of the Creative Commons Attribution 4.0 International license.

### Functional analysis.

To obtain a functional overview of the M. florum genomes, the genes from the core genome and pangenome were classified into clusters of orthologous groups (COG) functional categories (40). Most (74.7%) of the core genes could be associated with a COG category, while most (66.7%) of the accessory genes could not. Overall, more than half (53.4%) of the COG associations belonged to the translation (J), carbohydrate metabolism (G), replication/recombination/repair (L), general function prediction only (R), and amino acid transport metabolism (E) categories. The core genome was significantly enriched (Fisher test with Bonferroni correction, *P* < 0.05) for functions related to translation (COG category J) compared to the frequency of this same category in the entire genome (Fig. 2C). The distribution of COG categories in the pangenome was more diversified, and no category was found to be statistically significantly enriched or deprived when all M. florum strains were considered. Genes linked to carbohydrate metabolism (mainly phosphotransferase system [PTS] components and β-glucosidases) and genome maintenance, mostly in restriction/modification systems (COG G and L categories, respectively) varied greatly in number, depending on the strains, although they were generally more abundant in the accessory genome (Fig. 2D).

### Genome organization.

**FIG 3:**
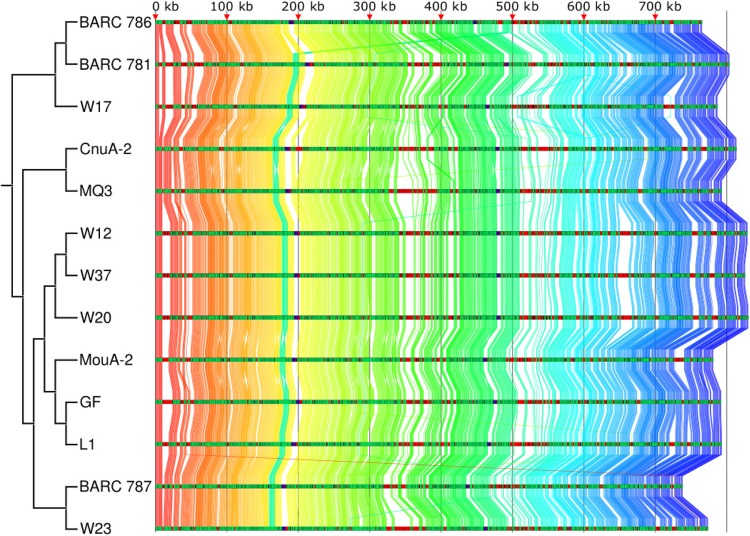
Core genome synteny of the 13 M. florum strains. Protein-encoding genes of the core genome are linked across all strains by using a color gradient based on the gene order observed in M. florum BARC 786. Each genome track is colored on the basis of core proteins (green), noncore proteins (red), and functional RNAs (purple). The topology of the distance tree is shown on the left.

We next analyzed the genomic organization in M. florum strains by characterizing the relative positions of the core genes. We found that although some rearrangements can be observed, the genomes are mostly syntenic with a large number of genes conserved between the different strains and variable regions located within the same relative genomic loci in every strain (Fig. 3). Furthermore, the gene order conservation (GOC) scores (41) of the core genes calculated for every combination of two strains averaged 0.98 ± 0.06, where a GOC value of 1 indicates that all core genes are found in the same order between two strains. We also noted the presence of large weakly conserved or nonconserved regions, consistent with the presence of genomic islands. Regions with at least three consecutive noncore genes were further studied to determine their potential status as genomic islands resulting from horizontal gene transfer. An average of 13.9 ± 3.4 such regions were identified in each genome, representing 19.4% of all noncore gene clusters. One such cluster, detected only in strains W37, W12, and W20, was located near the 660-kbp position and contained 16 hypothetical proteins, as well as 1 protein predicted to be part of a phage tail fiber. Using BLASTP, we searched for protein homologs in other organisms. Of those, 12 had hypothetical protein homologs in spiroplasmas and entomoplasmas, which are the genera closest to mesoplasmas among the members of the class Mollicutes. We also detected an 11-gene cluster ranging from position 14,058 to position 24,472 in strain BARC 781. According to the BARC 781 genome annotation, this cluster contained genes related to type IV secretion systems and a predicted mobile element protein, whereas in the other strains this region was enriched in PTS component proteins. Interestingly, the genomic location between translation initiation factor 2 and *dnaJ* was also found to be highly variable, even among closely related strains. Depending on the strain, this locus contained between 1 and 21 genes encoding proteins annotated as PTS components, hypothetical proteins, phage-related proteins, restriction-modification systems, and transcription regulators. These results suggest that horizontal gene transfer events have occurred within the M. florum species, shaping its genomic landscape.

### Transposon mutagenesis in M. florum L1.

In addition to comparative genomic data analysis, we also performed transposon mutagenesis to identify essential genes in M. florum L1. A collection of 2,806 mutants in which insertions occurred, on average, every ~280 bp across the genome resulted in the interruption of 430 of 720 genes (Data Set S4). No transposon was observed in the remaining 290 genes, which could be essential genes or simply have been missed given the transposon insertion density. The probability of observing no transposon insertions within a gene was calculated on the basis of the transposon insertion density and the length of each individual coding sequence (CDS) as previously described (16, 42), assuming that the probability of having *N* insertions in a gene of length *L* follows a Poisson distribution (Data Set S1). Although the transposon insertion density in our work is superior to what was reported in other gene inactivation studies involving Mollicutes (12, 43), the average probability that a gene could have been missed by chance in our experiment is ~10%, corresponding to ~69 genes for the entire M. florum L1 genome. However, this distribution is strongly skewed toward small genes, with more than half (37/69) of the potentially missed genes having a length of <400 bp. For simplicity, all 290 genes that were not interrupted by a transposon were nonetheless considered putatively essential despite these limitations.

10.1128/mSystems.00198-17.4 DATA SET S4  Transposon insertion sites in *M. florum* L1 in GFF3 format. Download DATA SET S4, XLSX file, 0.1 MB. Copyright © 2018 Baby et al. 2018 Baby et al. This content is distributed under the terms of the Creative Commons Attribution 4.0 International license.

**FIG 4:**
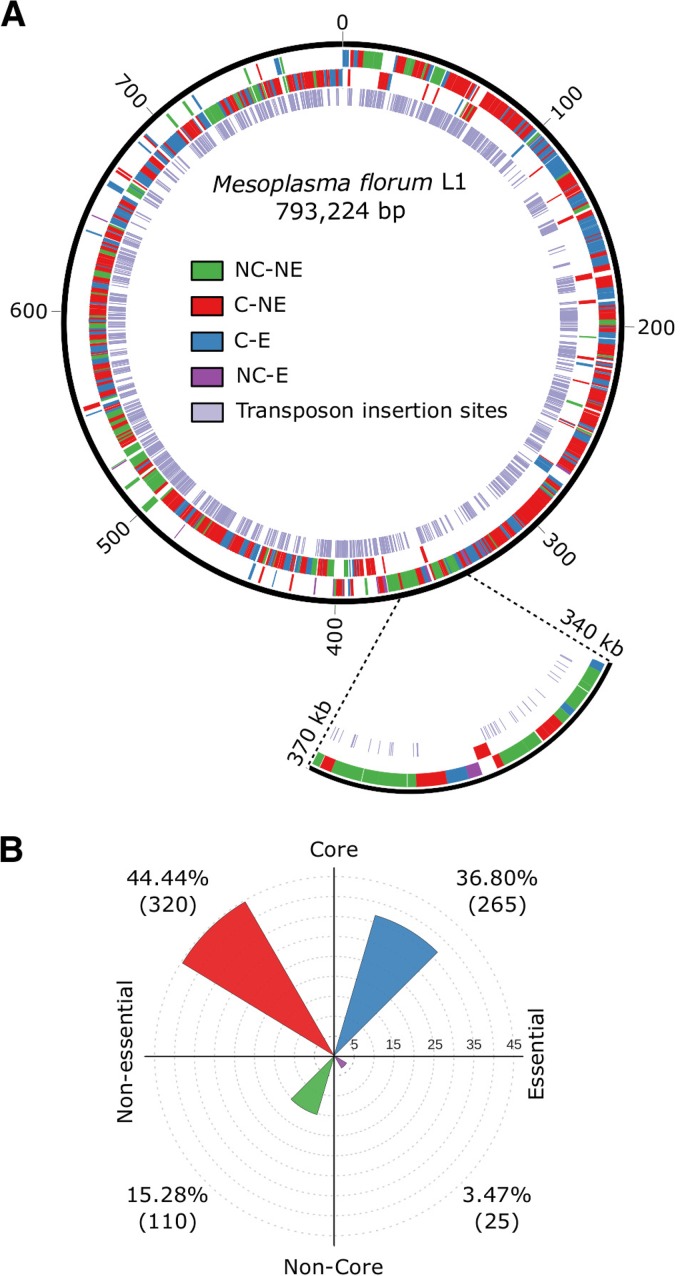
Overview of M. florum L1 genomic landscape based on gene conservation and essentiality. (A) Classification of M. florum L1 genes shown by a color representing core (C), noncore (NC), essential (E), or nonessential (NE) genes with plus strand genes in the outermost layer and minus strand genes in the middle layer. Transposon insertion sites are also in the innermost layer. The 340- to 370-kbp region is enlarged to show an example of a locus containing all types of gene categories. (B) Gene distribution across the different categories.

The gene interruption and conservation data for the L1 strain were combined to identify genes that are most likely to be important for M. florum (Fig. 4A). Most of the genes presumed to be essential in the L1 strain on the basis of transposon mutagenesis were also conserved across all M. florum strains, with only 25 that were not associated with the core genome (Fig. 4B). A putative function could be attributed to only 5 of these 25 noncore but essential genes, of which 4 could be associated with a COG category and the remaining 20 were annotated as hypothetical proteins (Data Set S1). The 110 genes interrupted by transposons and absent from the core genome represent interesting first-step candidates for genome streamlining.

### Potential scenarios for M. florum genome reduction.

**FIG 5:**
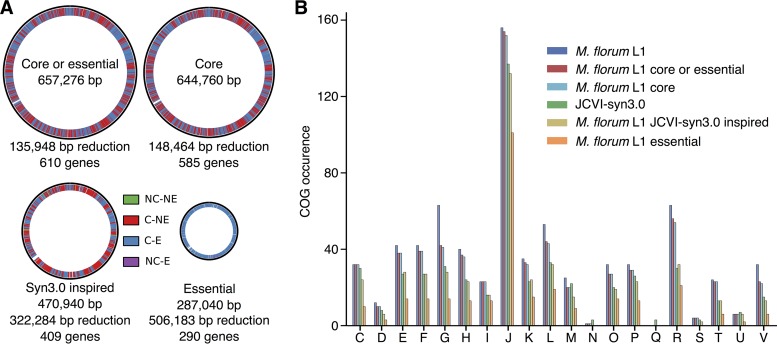
Genome reduction designs for M. florum L1. (A) Representation of four different versions of reduced M. florum L1 genome based on gene conservation, function, and essentiality. Genes are shown by a color representing core (C), noncore (NC), essential (E), or nonessential (NE) genes. In each case, the number of deleted bases is shown and corresponds to the sum of the lengths of the CDSs of the deleted genes. (B) Number of protein-encoding genes in each COG categories found in the different designs. The COG categories are as described in the legend to Fig. 2.

Three reduced versions of the M. florum L1 genome were designed on the basis of the gene conservation and transposon mutagenesis data (Fig. 5A). The first scenario excluded all noncore genes that were also interrupted by a transposon (610 genes remaining), the second contained only the core genes (585 genes from the 546 conserved gene cluster families in M. florum L1), and the third included only the putatively essential genes (290 genes). Both rRNA loci were included in these genome configurations, although only a single copy could be sufficient to sustain growth (2, 44). In each scenario, the approximate genome size was estimated by removing the CDSs of the candidate genes, while all non-CDSs were kept since most of the promoters and regulatory sequences in M. florum remain to be identified. A fourth genome reduction strategy was prepared by including all of the M. florum L1 protein-coding genes having an ortholog in M. mycoides JCVI-syn3.0, currently the closest approximation of a minimal genome. Interestingly, orthologs were identified for 401 of the 585 M. florum core genes. The M. mycoides JCVI-syn3.0-inspired genome reduction approach also included 5 genes identified as nonessential and noncore, while 57 genes marked as essential in M. florum L1 were absent.

We compared the COG category occurrences in the genome reduction strategies, as well as in the original M. florum L1 and M. mycoides JCVI-syn3.0 genomes (Fig. 5B). Globally, the proportions of the COG categories were similar in the genome reduction scenarios. Genes related to translation (COG category J) were more represented than any other. Genes related to lipid metabolism (I) made up the second most conserved category, although no gene predicted to perform lipid degradation or elongation was detected in M. florum. This category instead contained multiple acyltransferases, which could be used to anchor lipoproteins to the membrane (35), as well as a number of NADH and short-chain dehydrogenases. Overall, the carbohydrate metabolism (G) category contained the largest number of accessory genes since even in the most conservative proposition, 33.8% of these genes were identified as dispensable. Of those, 72.7% were PTS transport components or 6-phospho-β-glucosidases whose functions were duplicated.

## DISCUSSION

Minimal cells constitute powerful tools to better understand the fundamental components and the basic mechanisms that support life. The first approximation of a minimal gene set was recently provided with the creation of M. mycoides JCVI-syn3.0 (2). Technical advances now also enable the exploration of the M. florum minimal genome. The development of *oriC*-based plasmids and antibiotic selection markers (27) constituted the basic steps that led to the whole-genome cloning of M. florum in yeast (29). This was followed by the establishment of a genome transplantation protocol for M. florum and by the investigation of the impact of phylogenetic distance on this procedure (28). M. florum is therefore a bona fide candidate for genome reduction. However, this raises a few questions. Which genes should be removed to obtain a minimal M. florum genome? Given their phylogenetic proximity, would a minimal M. florum genome differ from or be equivalent to the minimal M. mycoides JCVI-syn3.0 genome? What could be learned by creating minimal genomes based on different cell chassis?

Two different approaches, comparative genomics and random transposon mutagenesis, were used to determine the gene composition of a putative minimal M. florum genome. The former exposed genes important for the survival of M. florum in its natural habitat, whereas the latter revealed the genes likely to be essential under laboratory conditions. Through the analysis of 13 different strains (Fig. 1), we determined the composition of the M. florum core genome and explored the diversity of its pangenome (Fig. 2). Although some strains were isolated from distant sites (Fig. 1A) and from different plants or insects (see Table 1), a total of 546 different protein-coding gene clusters, out of an average of 688 ± 23 per strain, were found to compose the core M. florum genome. Random transposon mutagenesis of strain L1 predicted a total of ~430 dispensable and ~290 putatively essential genes under laboratory conditions. It is possible that the relatively low transposon insertion density (on average, one insertion every ~280 bp) spared a small number of genes simply by chance, which would result in the inclusion of a few dispensable genes in the minimal genome. However, this is unlikely to significantly affect our general conclusions about which genes should be deleted first during an eventual reduction of the M. florum genome. Generating additional transposon insertion mutants would, however, increase the precision and confidence level of these predictions, especially for small genes.

Combining comparative genomics and transposon mutagenesis data can provide contrasting perspectives on which genes should be included in a minimal M. florum genome. While the 585 core genes could be expected to be sufficient for the survival of M. florum L1, ~25 noncore genes are expected to be essential according to our transposon mutagenesis of M. florum L1 (Fig. 4B). A minimal genome design based on conserved genes only is thus highly unlikely to produce a viable cell. This can be explained by the differences in the growth conditions and evolutionary pressures experienced by M. florum in the environment compared to laboratory settings. In fact, a majority, 320 (55%), of the of the 585 M. florum core genes are not essential in rich medium (Fig. 4B). An alternative scenario that includes only the 290 putatively essential genes is also questionable, as synthetic lethality is likely to occur and result in a nonviable minimal M. florum genome. This interpretation is supported by the fact that initially proposed minimal M. mycoides genome designs based on transposon mutagenesis and other literature-based knowledge were not viable (2). Preservation of both the core and essential genes would remove a total of 110 genes, which has a reasonable chance of success but would most probably remain far from the minimal genome composition.

Another possibility is to infer the minimal M. florum L1 genome on the basis of M. mycoides JCVI-syn3.0. A total of 409 M. florum L1 genes have homologs in M. mycoides JCVI-syn3.0. Of these, 404 are part of the M. florum L1 “core or putatively essential” gene set. Since all of the genes present in M. mycoides JCVI-syn3.0 are essential or have a strong impact on cell fitness, this reveals interesting differences between these organisms. Despite their phylogenetic relatedness, 69 gene families are found only in M. mycoides JCVI-syn3.0. Conversely, 57 putatively essential M. florum L1 genes have no homolog in M. mycoides JCVI-syn3.0 (Data Set S1). It is possible that some of these genes perform equivalent functions although their sequences differ significantly. However, a majority of these M. florum L1 (~61%) and M. mycoides JCVI-syn3.0 (~54%) genes are annotated as encoding putative or hypothetical proteins with no clear function, making further investigations more difficult. This highlights our current inability to unambiguously assign functions to a large number of genes and to analyze cell physiology by using a truly functional perspective, which constitutes a major challenge for biology. Genome scale *in silico* models (45) would constitute an attractive tool to help organize, refine, and compare the available information on minimal genomes. Nevertheless, a scenario emerging from this comparison would be to combine the 57 putatively essential genes found only in M. florum L1 to the 409 genes that have a homolog in M. mycoides JCVI-syn3.0. This would likely represent a better approximation of a minimal M. florum genome, given the data currently available. This also implies that the genome-reduced versions of these two organisms would, in large part, be similar but still differ despite their phylogenetic relatedness.

What could be the conceptual nature of the differences observed between M. mycoides JCVI-syn3.0 and the proposed M. florum minimal genome? In principle, the minimal genome can be divided into three categories, a hard, a semihard, and a soft minimal genome. The hard minimal genome includes genes encoding functions that are essential and performed in a similar fashion across different strains or species (i.e., genome replication, protein synthesis, etc.). The semihard category contains functions essential for any organism but for which alternative genes or strategies are possible to fulfill the same requirement. For instance, different gene families can ensure the same functions, as exemplified by nonorthologous gene displacement (46). The soft minimal genome is, on the other hand, composed of genes that are crucial in a given organism or environment but not necessarily in others. The availability of particular nutrients in the environment or the presence of a particular gene that affects the essentiality of other genes represents a possible factor affecting the soft minimal genome. The differences between M. florum L1 and M. mycoides JCVI-syn3.0 should vastly reside in either the semihard or soft minimal genome category. Since the semihard minimal genome of phylogenetically closely related bacteria is expected to be relatively small, the soft minimal genome is more likely to explain the distinctions between minimal M. florum L1 and M. mycoides JCVI-syn3.0 genomes. Indeed, the gene composition of these strains derives from data obtained in rich but slightly different media. Transposon mutagenesis of both strains in a set of different media would presumably lead to the identification of many environment-specific essential genes.

In conclusion, although the technology needed to build entire genomes is now accessible, synthetic genomics is increasingly limited by our understanding of cell functioning. A significant fraction of genetic components are still poorly characterized, even in the most thoroughly studied organisms. Because of their lower complexity, minimal genomes offer a remarkable opportunity to investigate the most fundamental cellular functions that support life. Furthermore, the construction of minimal synthetic chromosomes will facilitate the generation of several genome versions that could help better define the rules governing genome organization. The use of minimal cells will also facilitate the establishment of comprehensive whole-cell models, which is currently hindered by excessive biological complexity. These models could become powerful tools to predict cell behavior and to create synthetic genomes (47). Overcoming these important challenges will constitute a stepping stone toward the rational design and programming of complete genomes.

## MATERIALS AND METHODS

### Culture conditions and molecular biology methods.

M. florum strains were grown at 34°C in ATCC 1161 medium (27). Genomic DNA (gDNA) extraction was performed with the Quick-gDNA MiniPrep kit (Zymo Research) for the preparation of Illumina libraries and with Puregene Yeast/Bact. kit B (Qiagen) for PCR-free Sanger sequencing and Pacific Biosciences libraries. Extractions were made in accordance with the manufacturer’s instructions, except that M. florum cells were washed in a resuspension buffer (8 mM HEPES, 272 mM sucrose; pH 7.4) prior to gDNA extraction.

### Genome assemblies and annotation.

Genomes were assembled as previously described for the M. florum W37 genome (26). For each strain, two Illumina libraries were prepared, one with 200- to 250-bp inserts and the second with 450- to 750-bp inserts, both sequenced in paired-end reads of 144 and 100 bp, respectively. Pacific Biosciences RS libraries (obtained by C_2_ chemistry) with inserts of >5 kbp were also prepared. Error correction of the Pacific Biosciences reads was performed by using the Illumina reads, and all sequences were subsequently assembled with Roche gsAssembler version 2.6 and Ray version 2.1.0 (48). The two assemblies were merged and manually inspected before being completed (or completely scaffolded; Table 1) by Sanger sequencing of selected PCR products (the primers used are available on request). PCRs were performed with VeraSeq DNA polymerase (Enzymatics). The routine PCR conditions used were 30 s at 95°C; 30 cycles of 10 s at 95°C, 30 s at the appropriate annealing temperature, and 30 s/kbp at 72°C; and 2 min at 72°C. PCR products were purified by solid-phase reversible immobilization bead capture with Agencourt AMPure XP magnetic beads (Beckman Coulter, Inc.) (49). Sanger sequencing reads were generated by the Plateforme de Séquençage et de Génotypage of the Centre de Recherche du Centre Hospitalier de l’Université Laval. Pacific Biosciences sequencing was performed by the Yale Center for Genome Analysis and at the Centre d’innovation Génome Québec et Université McGill. All of the genomes were annotated with the RAST server (50) and FIGfam Release 70.

### Comparative genomics and functional analysis.

The annotated genomes were analyzed with get_homologues (51) (v.2.0) to identify the core genome and pangenome by the COGtriangle (37) method for bidirectional best-hit determination. The comparison of the M. florum strains and M. capricolum subsp. capricolum ATCC 2734 (NCBI RefSeq accession no. NC_007633.1) to identify the protein set used for the phylogenetic tree creation was performed by the same method, as was the comparison of M. florum L1 and the synthetic bacterium Syn3.0 (GenBank accession no. CP014940.1). Genome synteny was determined by using the core protein cluster coordinates and visualizing their locations across all of the genomes by using the GMV genome browser (v.1e-93) (52). GOC scores were determined by using the core genes as previously described (41). Genes separated by fewer than five core genes were considered contiguous (41). For the functional analysis, the latest COG database (53) was downloaded from the NCBI ftp website (ftp://ftp.ncbi.nih.gov/pub/COG/COG2014/data/). The proteins predicted in the 13 M. florum genomes were compared to the database with BLAST (54). The results were filtered on the basis of the E value with a stringent threshold of 1e-10. Available conversion tables from the NCBI ftp website were used to convert matching proteins to COG identification numbers to functional categories. Enriched categories were determined with the Fisher exact test and Bonferroni correction for multiple tests by comparing the frequency of genes in a COG category in the M. florum core or accessory genome with that in the entire genome.

### Phylogenetic tree construction.

An alignment of the amino acid sequence of 412 conserved proteins of the 13 sequenced M. florum strains and M. capricolum was made with ClustalO (v.1.2.1) (55). Unaligned and low-confidence regions were removed with Gblocks (v.0.91b) (56) to produce a sequence matrix of 138,476 amino acid sites. Both phylogenetic trees (Fig. 1B and C) were made from this alignment with SeaView (v.4.6.1) (57). The distance tree was generated by neighbor joining with BIONJ (58) and the Kimura distance model (38). The maximum-likelihood tree was generated with PhyML (v3.0) (39) and the LG evolutionary model (59). Bootstrap values were calculated by using 100 regular bootstrap replicates.

### Transposon mutagenesis.

Tn*5* transposomes were assembled *in vitro* with EZ-Tn*5* transposase (Epicentre) without Mg^2+^ as recommended by the manufacturer. The transposon DNA was obtained from the digestion of the pTT01 plasmid with restriction enzyme PvuII and conferred tetracycline resistance. The transposomes were transformed via electroporation as described by Matteau et al. (27) with a voltage of 2.5 kV. Insertion mutants were selected on ATCC 1161 solid medium supplemented with 15 µg/ml tetracycline. Colonies were picked as they became visible, with certain mutants growing more slowly than the parental strain. The transposon insertion site was determined by Sanger sequencing of gDNA. Genes that contained at least one transposon insertion were considered nonessential.

### Data availability.

The complete genome sequences and annotations of the 11 newly sequenced M. florum strains are available in GenBank under the following accession numbers: BARC 781, CP022511; BARC 786, CP022510; BARC 787, CP022514; CnuA-2, CP022513; GF, CP022509; MouA-2, CP022508; MQ3, CP022512; W17, CP022507; W20, CP022506; W23, CP022505; W12, CP022432. The comparison of NCBI annotations and gene predictions used in our study for strain L1 is presented in Data Set S1. Gene predictions for the 12 other M. florum strains can be found in Data Set S2. The composition of the M. florum COG can be found in Data Set S3. The transposon insertion sites observed in M. florum L1 are provided in Data Set S4.
